# Cadmium-Rich Plant Powder/PAN/PU Foams with Low Thermal Conductivity

**DOI:** 10.3390/polym14142893

**Published:** 2022-07-16

**Authors:** Wenying Tang, Jin Sun, Jie Tang, Zheng Chen, Yidong Shi, Ruifang Zhao, Yuanzhang Jiang, Lin Tan

**Affiliations:** 1 Sichuan Province Fiber Inspection Bureau, Chengdu 610015, China; tangwy1028@163.com (W.T.); scxjsunjin@foxmail.com (J.S.); michael.dore@163.com (Z.C.); 2 Key Laboratory of Leather Chemistry and Engineering of Ministry of Education, College of Biomass Science and Engineering, Sichuan University, Chengdu 610065, China; shiyidong@scu.edu.cn (Y.S.); tanlinou@scu.edu.cn (L.T.); 3 Sichuan Huafang Yinhua Co., Ltd., Suining 629200, China; tlly94619@126.com

**Keywords:** cadmium-rich plant, composite foams, low thermal conductivity, high cadmium immobilization rate

## Abstract

Treating and utilizing heavy metal enriched plants have become growing problems. In this work, a series of composite foams were made from the powder of Cadmium-rich plant, polyacrylonitrile (PAN) and polyurethane (PU). Test results indicated that the addition of plant powder can not only increase the specific surface area, but also improve the apparent density and thermal stability of the foams. Besides, compared with the foam without plant powder, the powder-added foams exhibited a decreasing trend for thermal conductivity, and the minimum was 0.048 w/(m·k), which indicated that the addition of plant powder can help to enhance the thermal insulation of composite foam. More importantly, the results of leaching experiment showed that the leaching rate of heavy metal cadmium in the composite foam with 50% plant powder content was as low as 0.14% after being immersed in the acidic (pH = 3) solution for 5 days, which implies that the foam materials are very safe. This study provides a new way to realize high value-added resource utilization of heavy metal-enriched plants.

## 1. Introduction

In May 2018, the Food and Agriculture Organization of the United Nations released *soil pollution: hidden reality* at the global soil pollution seminar, which pointed out that global soil pollution is becoming more and more serious [1]. Such a large area of soil heavy metal pollution poses a threat to food security, residential environment security, and ecological balance, and has become a global environmental problem [2,3,4]. Therefore, the treatment of soil heavy metal pollution is urgent.

Based on the mechanism, the existing remediation technologies for soil heavy metal pollution can be divided into physical remediation, chemical remediation, electrical thermal remediation, and phytoremediation [5,6]. Generally speaking, conventional methods like physical, chemical, and electrical thermal remediation technologies are costly and easy to cause secondary environmental pollution. In contrast, phytoremediation of heavy metal contaminated soil is not only low-cost, easy to operate, and environmental, but also can improve the soil quality with carbon sequestration, making it a popular remediation method [4,7]. Usually, phytoremediation utilized plants such as mustard, amaranth and so on that can absorb and fix heavy metals in their cells and tissues to reduce the metal concentration in the soil [8,9,10]. As well, it has been widely used in the remediation of heavy metal polluted mining areas and farmlands [11]. Despite the advantages phytoremediation technology exhibited, it brings a new problem—how to deal with biomass containing heavy metals.

Normally, the heavy metals in these stabilized by plants need to be isolated from the material cycle of the ecosystem, treated separately, and can no longer be released into the environment and ecosystem. At present, there are several ways to utilize remediation plants including biomass power generation [12], biofuel production [13], and building materials [14]. Biomass power generation refers to burning the remediation plants, and then using the heat generated by combustion or high-pressure steam for power generation. The centralized combustion of waste biomass into residues (fly ash and pulverized coal slag) can greatly reduce the volume and weight of waste in a short time [15]. As an important part of sustainable fuels, biofuels can effectively alleviate this environmental problem by converting waste biomass into solid, liquid, or gaseous fuels [16]. In addition, the heavy metal enriched remediation plants can be burned into ash and used as building materials after solidification, which not only reduces the cost of building materials, but also is a constructive step towards the management of hazardous waste [17]. Although the aforementioned methods provide ways for the resource utilization of biomass containing heavy metals, there are also some shortcomings. Table 1 lists the advantages and disadvantages of the above resource utilization methods. At present, there is a relative lack of research on biomass resource technologies for heavy metal-containing remediation plants, which largely hinders the engineering application of phytoremediation technologies [18]. Therefore, there is still a need to develop technologies and pathways for resource utilization of heavy metal remediation plants in order to promote the development and application of technologies.

In recent years, studies have revealed that new foams such as synthetic polymer foams, carbon foams, chitosan foams, and rhamnolipid foams have good adsorption effects on heavy metals. Among them, most of the studies on polymeric foams were prepared using polyurethane as the raw material [20,21,22].

Combined with the current problems and development trends, in this study, we prepared composite foams by mixing heavy metal remediation plants powder and polymer-carriers through a facile vacuum freeze-drying method. The test results revealed that the addition of plant powder can increase the specific surface area and apparent density of the foam, and thus enable low thermal conductivity. Meanwhile, even soaked in the acid solution for 5 days, the Cadmium immobilization rate of foam with 50% plant powder can still maintain >99.8%, indicating excellent safety. This study provides some new ideas for realizing the utilization of high value-added resources of heavy metal enriched plants.

## 2. Materials and Methods

### 2.1. Materials

*N*,*N*-Dimethylacetamide (DMAC), *1*,*4*-dioxane, ethanol, disodium hydrogen phosphate, ammonium chloride solid, H_2_O_2,_ nitric acid, and sulfuric acid were purchased from Chengdu Kelong Chemical Reagent Company. Thermoplastic polyurethane (TPU) was purchased from Shanghai Hiend Polyurethane Inc. and Polyacrylonitrile was purchased from China Petrochemical Corporation.

### 2.2. Treatment of Heavy Metal Enrichment Biomass

*Grain amaranth* is a heavy metal enrichment plant with fast growth, large biomass, easy cultivation, and strong environmental adaptability. It has been widely used in the remediation of cadmium-contaminated agricultural soil [23].

In this paper, the whole plant of *Amaranthus hypochondriacus* L. (APL), a commonly used engineering remediation plant for heavy metal pollution in soil, was selected as the research object. The APL was cultured in soil with a Cd content of 100 mg/kg, and the plants were harvested at a height of about 1 m after three months of growth. Before use, the plants were washed with distilled water, dried in a vacuum oven at 70 °C to constant weight, and then crushed into small particles by a multi-functional crusher (xqm-1sp 2, Nanjing University Instruments Co., Ltd., Nanjing, China), screened by 100-mesh sieve, and further processed into ultra-fine powder by a ball mill. A total of 0.1 g plant powder was added into the solution of 1 mL HCl, 4 mL HNO_3_, 1 mL HF and 1 mL H_2_O_2_, and digested in a digester. The content of cadmium in amaranth plant powder was determined by an Inductively coupled plasma mass spectrometry ICP-MS instrument (Nexlon 300, PerkinElmer, Waltham, MA, USA) and the average content of cadmium in the powder was 126.49 mg/kg [24].

### 2.3. Preparation of the Composite Foams

TPU is an elastomer, which has the advantages of excellent flexibility, wear resistance, tear resistance, and chemical resistance. It has become one of the six major synthetic materials in the world, and has been widely used in industries in different forms such as fiber, elastomer, adhesive, and coating [25].

PAN is a common raw material for processing fiber and membrane materials [26,27]. It is cheap and of good heat resistance and corrosion resistance (especially for some acidic substances such as hypochlorous acid, hydrogen peroxide, and general organic reagents) [27]. However, PAN fiber has low strength and poor elasticity, and wear resistance.

Based on the comprehensive consideration of the mechanical properties of the composite and the previous report, the mixing ratio 1:3 (PAN:PU) was selected, and the group with the best properties was used as the carrier of the composite [28].

**Figure 1:**
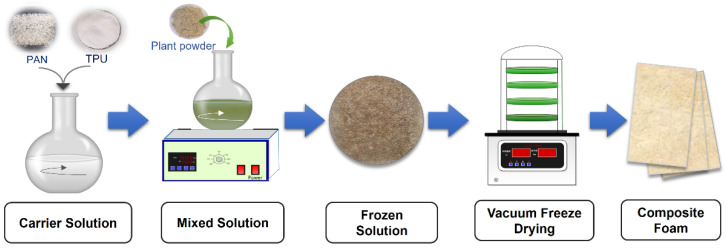
Preparation of the composite foams.

The preparation process of the foams was demonstrated in Figure 1. To reduce the corrosion of the vacuum drying equipment, *1*,*4*-dioxane was selected as the solvent. PAN and PU were firstly dissolved in *1*,*4*-dioxane and then the solutions were mixed and stirred to give a PAN/PU solution. Then, 0 wt%, 10 wt%, 20 wt%, 40 wt%, and 50 wt% of the total solid mass fraction of the plant power were added to the solution. After 1 h stir, the solutions were introduced into a pre-frozen mold and put into a freezer. Then the frozen solutions were lyophilized to prepare foam samples, namely, Foam-0, Foam-10, Foam-20, Foam-40, and Foam-50, respectively.

### 2.4. Characterization

#### 2.4.1. Basic Performance Test

The microstructure surface morphology of various composite foam samples was analyzed by a scanning electron microscope (SEM, SU3500, Hitachi, Japan) at an accelerated voltage of 15 kV. 

The thermal degradation performance of composite foam was tested by a thermogravimetric analyzer (DTG-60, Shimadzu, Japan) under N_2_ flow (50 mL/min) from 30 to 700 °C at a rate of 10 °C/min to analyze the melting temperature, glass transition temperature, and enthalpy change temperature and polymer compatibility of each composite foam. 

A contact angle tester (Harke-SPCAX1, Beijing, China) with a digital camera was applied to observe the water contact angle (WAC). 

The apparent density of the foams was tested and calculated according to GB/T 6342-1996. 

A surface area and pore size analyzer (Gemini VII 2390, Micromeritics, Atlanta, GA, USA) was used to investigate the specific surface area (BET), pore volume, and the pore size of the foams.

#### 2.4.2. Thermal Conductivity Test

After cleaning, the samples were cut into circles with diameters of 12.7 mm, and after being sprayed with graphite on the surface, they were measured with a thermal conductivity meter (LFA467 HyperFlash, Bavaria, Germany) to obtain the thermal conductivity. The test temperature range is 25 to 100 °C, the lamp voltage is 150 V, the sampling point is 2000, and the thermal diffusivity model is standard + pulse correction.

The thermal diffusivity of the samples can be calculated according to Equation (1).

(1) $$a=\frac{0.1388\times d^{2}}{t_{50}}\;$$

where *a* is the thermal diffusivity; *d* is the thickness of the sample; *t*_50_ is the half heating time, which is the time required for the upper surface temperature (detector signal) of the sample to rise to half of the maximum value after receiving the light pulse irradiation.

Then the thermal conductivity of the foams could be calculated according to Equation (2).

(2) $$\lambda \left(T\right)=a\left(T\right)\times C_{p}\left(T\right)\times \rho \left(T\right)$$

where *λ* is the thermal conductivity; *Cp* is the specific heat and *ρ* is the bulk density.

#### 2.4.3. Determination of Heavy Metal Stability of Composite Foams

To determine the Cadmium immobilization rate of Foam-50, 0.02 g of the shredded samples were put in aqueous solutions with pH of 3, 5, and 7, respectively. Then, the solutions were placed in a shaking incubator, mixed thoroughly, shaken slightly, and soaked for 5 days at room temperature. The leachate was then collected and analyzed by ICP-MS.

## 3. Results

### 3.1. Morphology Characterization

**Figure 2:**
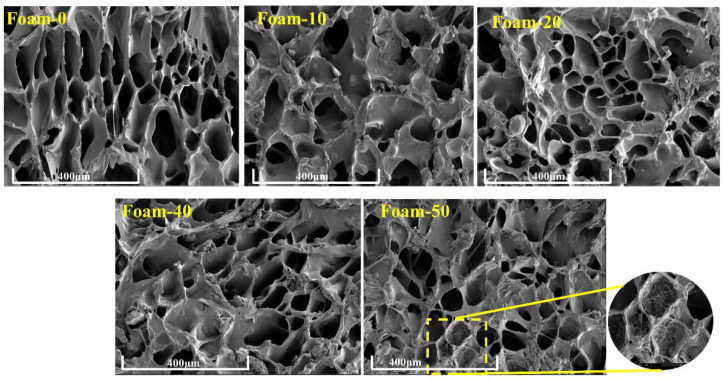
SEM images of different composite foams.

SEM was employed to observe the cross-sectional microstructure of the composite foams. As shown in Figure 2, the porous structure of the blank sample (Foam-0) can be clearly observed, and most of the pores are submicron-scale pores and open-cell morphologies, which is mainly due to the rapid evaporation of the solvent when the frozen solution was vacuum freeze-dried under low pressure and low temperature, leaving a solidified pore structure [29,30]. Compare with Fom-0, the pore structures of Foam-10 and Foam-20 are still relatively clear, but due to the increase of the viscosity of the solution, some adhesive closed-cell structures appear in the pores of foam, but there is no significant impact on the pore structure of the foam. With the continuous increase of plant powder solid content in composite foams, the adhesion of pore structure of composite foam became more and more serious, and more closed-cell appeared, especially in Foam-50 [31]. For low-density foam, the presence of closed pores may lead to shrinkage and defective foam cell structure [32]. The reason for this phenomenon is that the plant powder particles are not miscible with the polymer carrier solution, so the powder particles in the porous structure of foam seem to be wrapped by polymers and bonded together [33,34].

### 3.2. Specific Surface Area Analysis

In order to better explore the effect of powder solid content on the pore structure of foam, the specific surface area was determined. The results are listed in Table 2. Foam-0 was found to have the smallest specific surface area 0.1381 m^2^·g, and this value increased with the increment of the content of plant powder obviously. This is because, under uniform freeze-drying conditions, polyurethane foam obtained lower density, and might also form open pores, a more disordered structure. The data in Table 2 shows that the addition of plant powder has a significant impact on the specific surface area of composite foam, which is positively correlated, but the pore volume of foam is significantly reduced. It can be found that excessive powder content is also not conducive to the formation of pores in foam.

### 3.3. WCA Test

**Figure 3:**
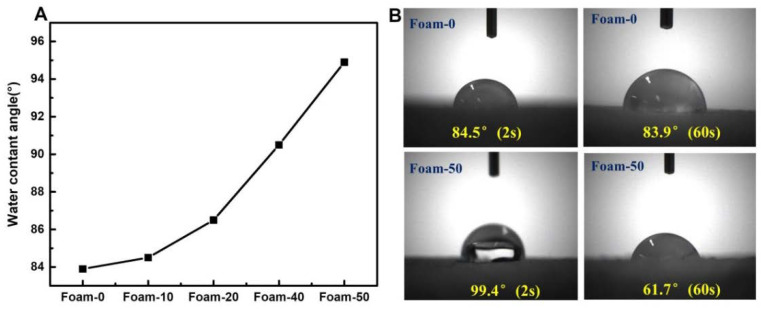
(**A**) Water contact angles of composite foams; (**B**) Water contact angles of Foam-0 and Foam-50 at 2 s and 60 s.

The WCA is mainly related to the surface microstructure and surface chemical properties, and is an important indicator to reflect the hydrophilicity and hydrophobicity of materials [35]. For the same material, the rougher the surface, the larger the contact angle. As shown in Figure 3, after the addition of plant powder, the surface became more hydrophobic, which might be caused by the higher roughness. Since the water contact angle of the water droplets on the surface of the syntactic foam changes greatly after staying for a period of time, Foam-0 and Foam-50, which have the largest water contact angle change after 60 s, are selected for comparison, as shown in Figure 3. When the contact time of the foam and water increased to 60 s, the WCA of Foam-0 decreased by only 2.1°, indicating that the water absorption of Foam-0 was relatively stable, and the water contact angle changes little with the increase of time. However, the WCA of Foam-50 decreased by 32.7° at 60 s compared with that at 2 s, and changed from hydrophobic to hydrophilic, indicating that plant powder can enhance the water absorption of the composite foams. The reason for this phenomenon may be that more plant powder particles were added to the Foam-50, making the surface of Foam-50 rougher and surface tension larger. So, Foam-50 appeared more hydrophobic upon initial contact (2 s). However, with the increase of water contacting time, Foam-50 became more hydrophilic after 60 s because there were more hydrophilic groups that can better combine with water in plant biomass. By contrast, the surface of Foam-0 foam was very smooth, and the water contact angle changed little with the increase of time. The occurrence of this phenomenon may be because plant biomass contains a large amount of organic components such as cellulose, hemicellulose, and lignin, and the hydrophilic groups contained in these components can better bond with water molecules, thereby enhancing the water absorption of syntactic foams.

### 3.4. Thermal Property

**Figure 4:**
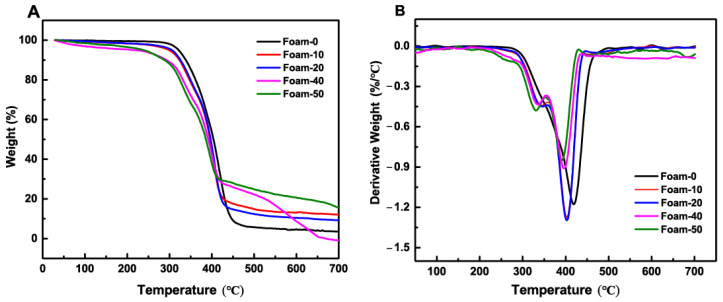
TG (**A**) and DTG (**B**) curves of composite foams.

A thermal stability analysis of composite foams was performed to investigate the effect of powder content on the thermal degradation of composite foams in the range of 30 to 700 °C. The TGA and their respective derivative (DTG) curves are shown in Figure 4. The thermal decomposition of the composite foams occurs in two successive stages in different temperature ranges: the first stage decomposition temperature is at 325 °C. This stage mainly involves the polymerization of the polyurethane foam and the production of volatile gases such as ammonia and carbon dioxide due to the breakage of the urea bonds, resulting in mass loss [3]. The decomposition temperature of the second stage is around 380 °C, whereas the decomposition temperature of Foam-0 is 420 °C, which is about 40 °C higher than that of the composite foam with plant powder, and the mass loss in this stage is mainly caused by the degradation of polyols into aliphatic compounds, aldehydes and volatilization of gases such as carbon monoxide [36]. After being heated at 700 °C, the mass loss of Foam-50 was the smallest, about 83.4%, and the mass loss of Foam-0 was the largest, up to 94.3%. Comprehensive analysis showed that when heated to 150 °C, the composite foams would decompose earlier than the blank sample. However, when heated to 400 °C, the mass loss of the composite foams decreased with the increase of powder content, and the mass loss rate of the foam was inversely proportional to the addition of the powder. This also shows that the plant biomass can effectively enhance the thermal degradation of composite foams at high temperatures above 400 °C.

### 3.5. Thermal Conductivity

Polyurethane foam is widely used in the field of materials, and thermal conductivity is also one of its most important properties [37]. According to Equation (2), the thermal conductivity of foam is mainly related to foam bulk density, thermal diffusivity, and specific heat. The relevant thermal conductivities of the above composite foams are listed in Table 3.

In Table 2, it can be found that the bulk density of composite foams increased gradually, but the thermal diffusivity and specific heat decreased. The overall thermal conductivity of composite foam showed a downward trend, which implied that the sequential addition of plant powder had a greater impact on the composite foam than the thermal solution.

**Figure 5:**
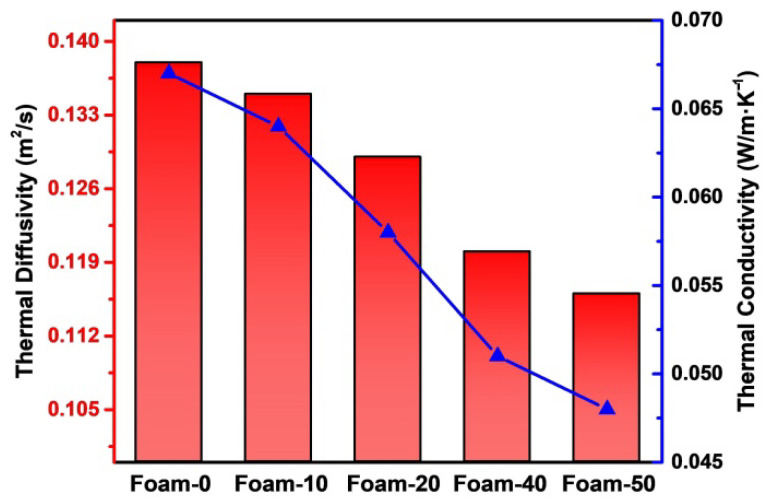
Thermal diffusivity and thermal conductivity of composite foams.

As shown in Figure 5, the thermal diffusivity and thermal conductivity of composite foams decrease step by step with the increase of plant powder. The largest thermal conductivity was demonstrated by Foam-0, which is 0.067 w·(m*k)^−1^. When the proportion of plant powder reached 50%, the thermal conductivity of Foam-50 is only 0.048 w·(m*k)^−1^, which is 28% lower than that of the Foam-0. The reasons for this phenomenon may come from two aspects. On the one hand, the addition of powder increases the closed-cell of the composite foams, and the heat conduction path becomes more complex, reducing the air heat flow generated by the composite foam receiving heat radiation [38,39]. On the other hand, researchers have demonstrated that high water content will reduce the performance of all foam materials, while the thermal resistance of biomass foam will be significantly reduced [35,40].

### 3.6. Heavy Metal Stability in Composite Foams

Cadmium is one of the most mobile and toxic heavy metals, and its fluidity will be significantly improved especially under acid conditions (pH < 6.5) [41]. In order to evaluate the safety of the material, Foam-50, with the largest plant powder content, was immersed in solutions with pH of 3, 5, and 7, respectively, for 5 days to test the leaching of heavy metal Cadmium. As shown in Table 4, the leaching rates of Foam-50 were very low (<0.2%). Even in the extreme acid environment (pH = 3), the Cadmium immobilization rate was still as high as 99.86%, indicating that the polymer solution carrier can well immobilize Cadmium. In addition, studies have shown that the adsorption of HAp/PVA cryogel immobilized on polyurethane (PU) foam follows the Langmuir isothermal model, and the maximum adsorption capacity for cadmium was estimated to be 47.7 mg/g [42]. This indicates that PU foam can not only fix heavy metals in plants containing heavy metals, but also have a certain adsorption on heavy metals in water, which also further illustrates the safety of the composite foam. Thus, it can be seen that composite foam has application potential as heavy metal adsorption material.

## 4. Conclusions

In this work, we made the Cadmium enriched plant into powder and then added the powder into polymer solutions to prepare composite foams. Such simple and easy-to-operate processing technology can further reduce the treatment cost of heavy metal-containing plants. The addition of the plant powder can effectively increase the specific surface area and the apparent density of the foams, which enables a lower thermal conductivity. Noticeably, the composite foam with 50% plant powder addition exhibited an extremely low leaching rate of Cadmium under acid solution, indicating the high safety of foams. This study provides new ideas and strategies for the resource utilization of heavy metal-enriched plant materials. The good performance and high safety of the materials have brought a wide range of application prospects for future industrial applications, and also achieved a win-win situation of environmental and economic benefits.

## Figures and Tables

**Table 1:** Recycling pathways and advantages and disadvantages of plants containing heavy metals.

Recycling Approach	Advantages	Disadvantages
Biomass Power Generation	Generate power/heat; reduce greenhouse gas emissions; recover precious metals from fly ash, have high economic value.	Produce secondary pollution (toxic air pollutants); extraction of heavy metals needs chelating agent, requires complex process and high cost.
Biofuel Production	Produce bio-oil, industrial ethanol, and other materials; further realize the reduction, harmlessness, and recycling of biomass containing heavy metals.	Require complex operation; the conversion efficiency is low; causes subsequent environmental problems.
Building Materials	Replace part of cement; reduce the cost of building materials; reduce CO_2_ and NO_x_ emissions under the condition of coordinated high-temperature disposal in cement kiln [19].	Heavy metals used in building materials need to be fixed; heavy metals may leach.

**Table 2:** Specific surface area and pore volume of the composite foams.

Samples	Specific Surface Area/m^2^·g
Foam-0	0.1381
Foam-10	0.3158
Foam-20	0.3179
Foam-40	0.3268
Foam-50	0.4392

**Table 3:** The relative parameters of thermal conductivity measured of composite foams.

Samples	*d* (mm)	*a* (mm^2^/s)	*ρ* (g/cm^3^)	*Cp* (J/g·K)
Foam-0	2.400 ± 0.043	0.138	0.253	1.861
Foam-10	2.500 ± 0.012	0.135	0.260	1.823
Foam-20	2.500 ± 0.068	0.129	0.266	1.690
Foam-40	2.800 ± 0.018	0.120	0.273	1.506
Foam-50	2.900 ± 0.021	0.116	0.300	1.386

**Table 4:** Cadmium immobilization rate of Foam-50 soaked in different pH solutions.

pH of the Solution	Mass of Foam (g)	Volume of Leachate (mL)	Cadmium Concentration in Leachate (μg/L)	Cadmium Leaching Rate (%)	Cadmium Immobilization Rate (%)
3	0.2	10	1.779	0.14	99.86
5	0.2	10	1.529	0.12	99.88
7	0.2	10	0.645	0.05	99.95

## Data Availability

Available data can be obtained from the corresponding author upon request.
